# Smart and Adaptive Architecture for a Dedicated Internet of Things Network Comprised of Diverse Entities: A Proposal and Evaluation

**DOI:** 10.3390/s22083017

**Published:** 2022-04-14

**Authors:** Shailesh Pratap Singh, Nauman Bin Ali, Lars Lundberg

**Affiliations:** 1 Ericsson Research & Development, 37133 Karlskrona, Sweden; 2 Blekinge Institute of Technology, 37179 Karlskrona, Sweden; nauman.ali@bth.se (N.B.A.); lars.lundberg@bth.se (L.L.)

**Keywords:** Internet of Things (IoT), smart and adaptive environment, artificial intelligence, multi-access edge computing (MEC), online gaming, 5G, architecture assessment, accounting, authentication, and authorization (AAA)

## Abstract

Advances in 5G and the Internet of Things (IoT) have to cater to the diverse and varying needs of different stakeholders, devices, sensors, applications, networks, and access technologies that come together for a dedicated IoT network for a synergistic purpose. Therefore, there is a need for a solution that can assimilate the various requirements and policies to dynamically and intelligently orchestrate them in the dedicated IoT network. Thus we identify and describe a representative industry-relevant use case for such a smart and adaptive environment through interviews with experts from a leading telecommunication vendor. We further propose and evaluate candidate architectures to achieve dynamic and intelligent orchestration in such a smart environment using a systematic approach for architecture design and by engaging six senior domain and IoT experts. The candidate architecture with an adaptive and intelligent element (“Smart AAA agent”) was found superior for modifiability, scalability, and performance in the assessments. This architecture also explores the enhanced role of authentication, authorization, and accounting (AAA) and makes the base for complete orchestration. The results indicate that the proposed architecture can meet the requirements for a dedicated IoT network, which may be used in further research or as a reference for industry solutions.

## 1. Introduction

Recent advances in 5G [1,2,3,4] and the Internet of Things (IoT) have demonstrated that the expectations and requirements for the next generation of communication systems can be defined as a network of heterogeneous devices, applications, sensors, access technologies, and stakeholders which come together for a common purpose. Diverse cyber-physical objects as part of the collective requirement shall have to be modelled together as part of a dedicated IoT network [5,6]. This network shall have to support heterogeneity among the different stakeholders and services [6,7]. Therefore, it is very challenging to perceive all the different requirements and the stakeholders that may come together for a common purpose at one point in time. Connecting a kind of device (such as a smartwatch, a health monitor, and other similar perceived IoT devices) to a network may not require connecting it to the internet comprising of a diverse set of entities. Rather a dedicated IoT network may require diverse users, devices, sensors, applications, communication service providers, and other different stakeholders coming together for a common purpose. An IoT network required for a use case such as online gaming would require a software defined network supporting a smart and adaptive environment to handle its various requirements. By virtue of its name, this internet or network of things is an evolving and heterogeneous entity [2,8]. Multiple research and standardization activities globally contribute to its evolution towards a multi-service adaptive network architecture [9] based on the representative use case families for 5G and IoT. Such representative use cases in the form of the enhanced Mobile Broadband [10] use case family are explored in this paper.

Existing research artifacts have analyzed and presented the importance of middleware or an agent for control, management, and orchestration in such a diverse and evolving IoT network [11,12,13]. These agents can be federated and distributed. Such a multi-agent system can bring value based on each agent’s context and as part of the IoT network. Existing literature describes how context awareness enhances the 5G multi-access edge computing reliability [14,15]. It is further stated that the exponentially increasing number of IoT devices and their diverse requirements would require dynamic orchestration which can be accomplished with virtualized and enhanced AAA (authentication, authorization, and accounting) in a distributed and intelligent fashion as network function virtualization in a software-defined network [16,17]. With the constrained environments and reduced interfaces for these IoT devices, their orchestration with authentication, authorization, and accounting also needs to be performed in an intelligent manner with a learning system as part of the ecosystem for understanding the usage and user patterns [18] and adapting the network behavior and capabilities accordingly.

The current IoT use cases are regarding a device or set of devices connected to a network from one vendor. However, a practical IoT setup would require a multi-vendor configuration. The number and frequency of vendors will change based on the evolving requirements in such a network. Therefore, there is a need to:

1. Describe the role of different communication service providers, applications, domain providers, and other service providers in such a setup.
2. Architect a system that can meet the requirements of the above scenario.
3. Evaluate the proposed competing architectures’ functional suitability, performance, and modifiability.

The primary purpose of this research is to identify and evolve a representative use case from the industry, get change scenarios from the domain experts, propose candidate architectures and perform a scenario-based software architecture analysis of the existing system and the proposed architectures. The evaluation is focused on the maintainability, functional suitability, and performance of the proposed architectures. Furthermore, the development effort for the candidate architectures under different scenarios was estimated using expert judgement. As expected, different architectures are suitable for different sets of requirements. However, it is evident from the analysis that the “smart AAA agent” fulfills the largest set of requirements and change scenarios, requires similar initial effort to implement and requires significantly less effort to maintain and evolve.

The Gaming Inc use case was chosen by the industry experts primarily for its business significance [19]. Furthermore, it meets the requirements of needing a smart and adaptive environment in a dedicated IoT network. This use case also contains the basic requirements of an IoT use case with different sensors, such as a heart rate sensor, facial expression sensor, vibration sensor, a gyroscope sensor, and other sensors required for providing a real-life experience to the gaming user.

The use case was further evolved during the interview and workshop with industry experts. We identified the additional requirements of multi-stakeholder support (i.e., a gaming user could freely choose their operator providing the high-speed bandwidth for the game). For example, a user should be allowed to have a gaming contract with the gaming company and should not be bound to choose a communication service provider for it. The gaming company should have a separate contract for the high-speed bandwidth with communication service providers. The user should also be able to get seamless gaming services over the different mediums of the internet. Other stakeholders can also provide content, devices, and sensors for the gaming company. There could be a separate service provider for additional services such as billing-as-a-service. This use case also requires autonomic network management [20] based on the stakeholder policies and the current context of the gaming user, such as their heart rate, geographical location, facial expressions, and many more. It also requires efficient monitoring of the entities in the dedicated IoT network for their quick assimilation and context-based changes.

The following are the main contributions of the paper:

C1. Described a representative industrial use case: Identification, exploration, evolution, and presentation of an industrial use case that is representative of a dedicated IoT network with major requirements as a part of enhanced mobile broadband spectrum and making it available for the research community. This is an important contribution since, as described above, the gaming domain will have significant future growth, and it presents unique challenges for a dedicated IoT network. Following a systematic approach (Section 3), we have further elaborated the use case in several fundamental ways, which include: the addition of a multi-stakeholder perspective, the ability to have different communications service providers and contracts, scalability to accommodate additional sensors, efficient monitoring and autonomic network management, and billing-as-a-service.
C2. Proposed candidate architectures: Two candidate architectures for addressing the challenges and meeting the requirements of the identified use case.
C3. Evaluated the proposed architectures: Scenario development and detailed scenario-based architecture analysis of the proposed candidate architectures and systems with leading domain and IoT experts. Moreover, expert-judgement based effort analysis of the candidate architectures.

The remainder of the paper is structured as follows: Section 2 briefly presents the related works. In Section 3, we describe our research methodology. Section 4 discusses the threats to validity in the study. Section 5 presents the study results in terms of the three main contributions (C1–C3) of the paper. Section 6 presents a discussion of the results and the updated architecture based on the evaluations. Section 7 concludes the study and describes possible future work.

## 2. Related Work

Dedicated IoT networks comprise diverse cyber-physical entities coming together for a common cause. An existing research paper [5] elaborates that there can be different physical and virtual objects in an IoT network and how the modeling of real life entities into a virtual object is a major challenge that needs to be addressed. Vlacheas et al. [6] discuss the major challenges and issues in a dedicated IoT network and enlists some such as the heterogeneity among connected objects and the unreliable nature of associated services. The authors also suggest a cognitive management framework, and exemplify it with a smart city model.

The mobile cloud gaming report [19] mentions the scope of cloud gaming as a big opportunity for 5G and IoT. We leverage on this report and evolve the gaming use case from the industry in discussions with experts by bringing in the multi-stakeholder and multi-service provider model. The rapid evolution of such networks with the evolution in technology and uses has also been discussed in the literature [2,8]. The role of middleware or an agent-based architecture has also been advocated by other researchers and experts [11,12,13]. Therefore, in this paper, we report the proposed candidate architectures having distributed agent-based architecture and their evaluation.

Furthermore, the need for context-aware dynamic behavior, including authentication, authorization, and accounting, has been discussed extensively in research artifacts such as [14,15,18]. A distributed, decentralized edge-/fog-/cloud-based architecture [21,22,23] has been discussed and evaluated for some IoT scenarios. However, there is a requirement of architecture and its evaluation for heterogeneous devices communicating over heterogeneous networks with various service providers coming together for a dedicated IoT network. Such an architecture also needs to consider the highly evolving nature of requirements and diverse stakeholders that shall come together as part of the network. In this paper, we identified and elaborated the needs for such a business use case, proposed and evaluated candidate architectures to fulfill the requirements of the case using systematic and rigorous architectural design and evaluation approaches.

Several reference architectures have been proposed for IoTs [24]. The most relevant for our work is the European Telecommunications Standards Institute’s Machine to Machine (oneM2M TS-0003) (ETSI—M2M https://www.etsi.org/technologies/internet-of-things, accessed on 18 January 2021) that provides a high-level security architecture. Their proposed three-layered architecture handles authentication, identity management, authorization, and security administration in the security functions layer. Our work complements this standard by adding an architecture that would allow multiple service providers to collaborate in a dedicated IoT network.

In the literature, several frameworks for developing IoT systems are proposed [25,26]. However, this is not the focus of our work. From the perspective of the current study, IoT systems leverage the features of a dedicated IoT (for an overview of existing research on authentication and authorization for IoT at the application layer, please refer to a review by Trnka et al. [27]). As part of our research the proposed smart AAA agent, and the static AAA agent distributed architecture for dedicated IoT networks will provide a baseline for the development of novel applications and user experience.

## 3. Methodology

**Figure 1:**
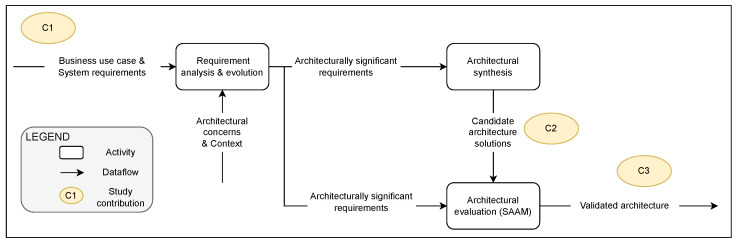
An overview of the research approach and contributions using Hofmeister et al.’s [28] approach for architecture design.

We now briefly describe our systematic approach to arrive at the three main contributions (as listed in Section 1). Our approach is based on Hofmeister et al. [28]’s general model of software architecture design. We have used their approach as it has synthesized five of the leading industrial approaches for architecture design. Figure 1 provides an overview of our approach and annotates the main contributions.

### 3.1. C1—Describing the Representative Use Case

We used two main sources to gather the requirements for a representative use case of a dedicated IoT network, which includes: industrial whitepapers (e.g., the TM forum for 5G monetization [29]) and interviews with various domain experts from the industry. We chose an online gaming use case from the “5G IoT lab” of a leading telecommunications vendor since online cloud gaming is recognized as a promising business opportunity [19] by the industry experts. The use case was evolved based on the interviews with four leading industry experts. These experts provided various perspectives of key stakeholders due to their experience and current roles. Brainstorming with the experts helped evolve the use case with multiple practical aspects in a real-world IoT scenario. These discussions helped evolve the use case beyond the boundary of one communication service provider and one gaming provider. It emphasized the role of different services, content, sensors, and devices that different stakeholders can bring about in such a system to make it a more meaningful, smart and adaptive dedicated IoT network. These interviews helped develop a multi-stakeholder and service provider model.

The interviews were structured as four 90-min, one-on-one workshops with the industry experts. We followed the guidelines collected by Runeson and Höst [30] to design and conduct the interviews. A detailed presentation of the use case from the ‘5G IoT lab’ was made as a baseline. This was followed by a discussion of limitation and improvement suggestions by the experts. All interviews were recorded and later transcribed. All improvement suggestions and scenarios identified by the analysis of the transcribed interviews were sent back to the experts for validation. Only one of the four experts added some additional reflections.

Then the validated input from all individual experts was consolidated. We used Zachman framework to break down all the requirements and policies for the dedicated IoT network use case in terms of context aware authentication, authorization, and accounting requirements, looking at AAA in an enhanced perspective taking care of the complete requirements. Zachman framework has been used in implementing enterprise architectures [31,32]. It uses primitive interrogatives what, how, where, who, when, and why to describe the desired system behavior [31,32]. Table 1 only exemplifies the corresponding dynamic AAA requirements based on the Zachman framework (the results of the study are reported in Table 2, where the color coding of the text corresponds to that of the basic interrogative).

The updated use-case and associated scenarios based on the consolidated input from all experts was again reviewed by all experts.

### 3.2. C2—Developing the Candidate Architectures

As suggested by Hofmeister et al. [28], we started with the business use case of enhanced AAA in a dedicated IoT network for gaming (derived using the approach described in Section 3.1). In the next step (see “requirement analysis & evolution” step in Figure 1), we analyzed these requirements for architecturally significant requirements (requirements that need to be considered when designing the system’s architecture). Domain experts were consulted (via interviews and workshops) to identify and prioritize quality characteristics of importance for the given business use case and the context. We used these quality characteristics to identify architecturally significant requirements by analyzing their impact on the ability of the system to fulfill them. Next, we performed architectural synthesis (see Figure 1), where we took decisions about the architectural styles, and specified the composition of the structural and behavioral elements of the systems. We consulted domain experts (workshop with an industrial chief software architect) and used our own experience for this synthesis. This resulted in two main candidate proposals: (a) static-rule-based agent and (b) a dynamic smart AAA agent.

### 3.3. C3—Architecture Evaluation

As the primary purpose of this research is to identify and understand the industry perspective for a dedicated IoT network and evaluate the existing and proposed candidate architectures and systems, we employ a scenario-based architecture analysis method (SAAM) [28,33,34,35]. SAAM has been extensively used for evaluating architectures in different domains [36,37,38].

The detailed workshop with the industry experts was conducted based on SAAM. Both the candidate architectures have been evaluated for the chosen scenarios representative of the key requirements of the dedicated IoT network bringing in the smart and adaptive environment. After assimilation of the inputs from the different experts a follow up workshop was conducted to ascertain the validity of the cumulative inputs from all of them. The main steps performed during the workshop were as follows:

1. The candidate architectures were explained to the experts.
2. Scenarios were developed based on the chosen use case as in Section 3.1 above.
3. Each scenario was evaluated for both the candidate architectures keeping into perspective the functional suitability, performance, and modifiability quality parameters.
4. Scenario interactions were discussed.
5. The transcripts of the workshop were shared with the experts for validation.

The duration of each of these workshops was around 90 min. A follow-up workshop was conducted after assimilation of all the inputs and evaluations for the final consensus from the experts.

**Evaluation goals:**
  Software and system architecture analysis was performed in this study, taking the major quality characteristics of functional suitability, maintainability and performance into consideration. Therefore, the following parameters based on the ISO 9126 and 25010:2011 standards [39] were used for evaluation. The product quality characteristics considered are: (1) maintainability, (2) functional suitability, and (3) performance. There definitely can be many other important quality characteristics such as security and reliability expected from a mature system. However, as the focus of this research paper is to provide candidate architecture for taking care of the disparate requirements of the different stakeholders coming together in a dedicated IoT network, we shall perform the evaluations on the decided quality characteristics.

The effort for setup and enhancements of the proposed candidate architectures under different scenarios is also evaluated as part of this study for analyzing the applicability and suitability of the architecture and system under different requirements. Effort estimation was conducted keeping the following into perspective: (1) initial setup, i.e., the upfront cost of moving from the current way of working to the candidate solution proposed in this paper, and (2) change scenarios, which encapsulate the expected changes with a likely impact on the architecture of the system.

Leading industry and domain experts were interviewed to discuss the scenarios and change scenarios and perform the scenario-based software architecture analysis of the candidate systems and architectures. A total of eight change scenarios were grouped into six groups and evaluated against the key parameters of maintainability, functional suitability, and performance. The proposed candidate architecture was also evolved with the help of an industry software architecture expert.

Effort analysis of two selected change scenarios was evaluated by a couple of expert program portfolio managers from the industry. These industry experts were chosen based on their expertise and familiarity with this use case and their prior involvement in similar tasks, to avoid any ambiguity and difference in understanding. For the effort analysis with the experts, the project management body of knowledge (PMBOK) [40] was taken as a reference and a detailed work breakdown structure was created for the two chosen change scenarios.

Further effort analysis has been performed, and the result is based on keeping the following external parameters constant:

1. Different requirements are well understood by the different stakeholders involved in the usability process.
2. Different team members’ technical competence is adequate for the job with minimum variance between the members.
3. Different team members’ domain competence is adequate for the job with minimum variance between the members.

This effort analysis is based on the following relevant estimation techniques as discussed and suggested by the experts and taken from the PMBOK [40]:

1. Three-point estimating (considering the best-case, most likely, and the worst-case estimates and combining in a beta-PERT distribution [41]).
2. Reserve estimating (with the contingency reserves for the risk of the known unknowns of the project).
3. Analogous estimating (utilizing the analogous measures for a similar set of activities).
4. Bottom-up estimating (using a work breakdown structure for the initial cost and change-scenario-based cost).

Our effort analysis result utilizes PERT (program evaluation and review technique) [41] which employs the following types of time involved in the effort for a task based on the three-point estimation:

1. Optimistic time (*o*) is the time based on the ideal availability of resources, and their ideal productivity. It is the minimum possible time required to accomplish an activity or task, assuming all circumstances are better than normal.
2. Pessimistic time (*p*) is the maximum possible time required for accomplishing a task taking the worst-case scenario into perspective.
3. Most likely time (*m*) is based on the best-case scenario assuming all circumstances behave as normal. It is the best estimate or most likely amount of time required to accomplish a task.
4. Expected time (*te*) is the estimated time for accomplishing an activity or task, taking into consideration that normally all circumstances do not fall in line as expected.

Therefore, the expected time *te* can be a weighted average of time with the most likely time getting four times the weight in comparison to the optimistic and the pessimistic time.

(1) $$te=\frac{o+4m+p}{6}$$

Assuming that there are *n* activities in a task, the total time estimated for a task shall be a summation of the expected times of the individual activities.

(2) $$TE=\sum_{i=1}^{n}te_{i}$$

This effort analysis is based on the initial effort and the effort for the two change scenarios selected for the analysis. The detailed work breakdown structure used for estimating effort is presented in Section 5.3 and the estimates are presented in Table 3.

Based on their expertise, familiarity with the use case, and the domain, six leading domain and IoT experts were selected as participants for this study. This list also contains a couple of program managers, with expertise of effort estimation for such large-scale systems. The profiles of these experts are briefly summarized below:

- Telecom and Internet of Things domain experts:
  Expert 1 (A Senior Specialist R&D at a leading Telecommunications vendor with 29 years of experience in Telecom and “Internet of Things” domains)
  Expert 2 (A Subject Matter Expert at a leading Telecommunications vendor with 31 years of experience in Telecom and “Internet of Things” domains)
  Expert 3 (A Chief Domain Architect at a leading Telecommunications vendor with 14 years of experience in software architecture and the Telecom domain)
- Software Architect:
  Expert 4 (A Chief Software Architect, Next Generation BSS at a leading telecommunication vendor with 19 years of experience in software architecture in the Telecom domain after doctorate degree)
- Program Managers:
  Expert 5 (A Portfolio Program Manager at a leading telecommunication vendor with 13 years of experience in the Telecom domain and project/program management)
  Expert 6 (A Portfolio Program Manager at a leading telecommunication vendor with 16 years of experience in the Telecom domain and project/program management)

## 4. Threats to Validity

We undertook several measures to mitigate the various threats to the validity of our approach. Such measures and the limitations of the study are briefly discussed below.

**Interviews:** Interviews conducted with different experts from one department could introduce bias. Therefore, the participants were chosen from different teams and departments. As the lead author is employed at a company from which the experts were chosen, extra precautions were taken to ensure no conflicts of interest (e.g., by not involving experts who report directly or indirectly to the lead author). Proper care was taken while conducting the series of interviews to let the participants provide their impartial feedback. These interviews varied from an hour to two for each session to allow ample time to explain the perspective and gather feedback. Several follow-up sessions were conducted for the queries from the experts. However, as all experts are from the same company and domain, perhaps the results indicate a Telecommunications vendor’s perspective.

**Workshops:** We held one-on-one structured workshops with the experts for the architectural design and evaluation. We made a detailed presentation of the material to each participant and collected their critique and improvement suggestions. For each workshop, the feedback was analyzed and incorporated into the study. Furthermore, the updates were discussed with the workshop participants in follow-ups. Workshops with groups of experts could have led to richer discussions and insights. However, it was practically difficult to book these experts for the same time slot.

**Effort estimation:** We consulted two project managers for estimating the development and maintenance effort for the candidate architectures. Both managers independently estimated the effort for the same tasks. This was conducted to increase the reliability of the estimates.

We used PERT as recommended by PMBOK. In addition, the experts are familiar with the method and use it for effort estimation for their regular work tasks as project managers. With the help of the experts, we also developed a detailed work breakdown structure to assist the task of estimation. We contend that detailed WBS and relying on an estimation method that the practitioners already use helps provide realistic estimates. Furthermore, PERT gives more significant weightage to the average values and thus reduces the outliers’ influence.

When estimating the effort, several parameters were considered constant, e.g., that the requirements are well understood by the different stakeholders involved in the development and that adequate technical competence and domain knowledge is available during the project. These assumptions (although likely to be violated in practice) are necessary to derive an estimate. Even with these limitations introduced due to the simplifying assumptions and the inaccuracy of the expert judgment-based effort estimates [42], we think that it is sufficient for a relative comparison.

**Architectural design:** Through the use of the Zachman framework, Hofmeister et al.’s model [28], and SAAM, we have used a systematic approach to architecture design in this study. The approach allowed us to identify the use case requirements, identify a subset of architecturally relevant scenarios, and develop and evaluate candidate solutions that can meet these requirements. However, the design decisions in the architecture are heavily influenced by the knowledge and experience of the experts. No systematic endeavor to consider multiple architectural styles and patterns reported in the literature was undertaken. This is not a considerable limitation of the study as the experts involved in the study are leading domain and architecture experts in the industry.

## 5. Results

### 5.1. C1—Use Case

**Figure 2:**
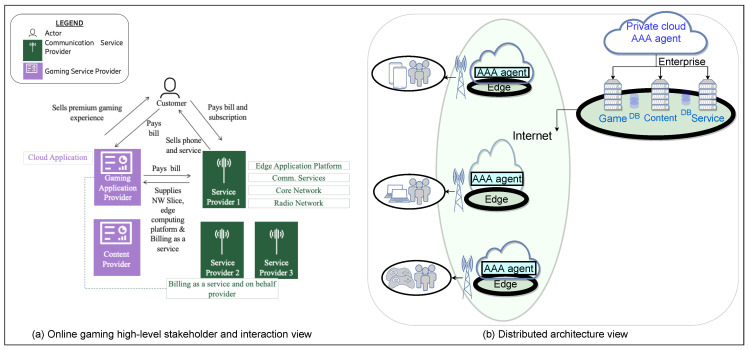
Online gaming- high level stakeholder and interaction view and the candidate architecture distributed view.

The scenarios for this study are based on the online gaming use case as depicted in Figure 2. It has been evolved and enhanced based on interviews (as mentioned in the Methodology Section 3) with the leading domain experts and introducing the multiple service provider model. Making such an industry lab use case available to the research community and its evolution is the foremost contribution of this research artifact. Following are the major contributions towards the evolution of the existing gaming use case to its current form based on the brainstorming sessions and workshops.

1. The multi-stakeholder view that goes beyond the current perspective of one communication service provider and one gaming company providing all the requirements of the dedicated IoT network for the gaming use case.
2. A gaming user cannot be bound to only one communication service provider or only one communication medium for playing the games.
3. There can be multiple communication service providers in a region or country and the gaming user should be able to play the game (with high speed) irrespective of the communication service provider as a gaming user has a contract with the gaming company and not them for the game.
4. The gaming company should have a separate contract with the different communication providers for a network slice with high bandwidth for its games.
5. Besides, a gaming company may require several other stakeholders to bring their contents, sensors, and devices into the gaming ecosystem.
6. There could be various other services, such as a billing-as-a-service which could be provided by one of the providers in the system.
7. Autonomic network management would be a significant requirement for the quick assimilation of all the different stakeholders to inter-work together as well as automatic network changes based on the different contexts of the gaming user and other stakeholders in the system.
8. Efficient monitoring of a large number of different stakeholders should also be a requirement in the evolving nature of the stakeholders and the frequent context changes taking place in the system.

As can be seen in Figure 2a, the provider comprises the Gaming Inc. application provider, sensor, device, and content provider. They keep sensitive customer data in their own cloud application and deploy only the core gaming application over the edge application platform provided by the service provider owing to security and confidentiality reasons. There can be more than one component (service provider) as depicted in Figure 2. Gaming Inc. buys a private network slice from the service provider(s), identified by S-NSSAI (single network slice selection assistance information) for a guaranteed high speed gaming experience as depicted in Figure 2a. The corresponding distributed architecture view in Figure 2b, depicts the similar multi-stakeholder view on the right. In the middle of Figure 2b, it depicts the multi-stakeholder view from the communication service provider and also different parts of the network, such that the multi “AAA agent” executing in a distributed manner on the edge of the communication network takes care of the requirements dynamically in the complete dedicated IoT network. This distributed “AAA agent” architecture enables a smart and adaptive environment which brings in some smart and adaptive features such as the following:

1. Assimilation of the different stakeholders in the evolving system.
2. Run-time-properties based adaptations, such as customer usage and his/her heart rate.

This multi “AAA agent” architecture also depicts an autonomic network management architecture, that intelligently adapts to the contexts and stakeholders in the network and smartly adapts the processing and its location based on the requirement. An efficient monitoring is also an important aspect in this network.

Billing as a service and billing on behalf can be services provided from one of the service providers in the network catering to the charging and billing of the Gaming Inc. Although the customer gets the bill with the Gaming Inc branding and pays the bill to the Gaming Inc, the system utilizes the services of the centralized billing as a service and billing on behalf of the provider for its billing requirements. Therefore, application providers focus on their expertise and offering and conduct charging and billing by the service provider. The different sensors and devices may also be provided by a different provider to the Gaming Inc. The customer on the top of Figure 2a has one contract with the communication service provider for the communication services such as data, voice, and messaging. The customer has a separate contract with Gaming Inc. for the gaming app and pays to them for the premium gaming services, devices, sensors, and experience.

Key high-level requirements from such a dedicated IoT network, identified from the whitepapers and refined based on interviews and discussions with the domain and IoT experts are listed below verbatim:

1. The dedicated IoT network should provide a seamless gaming experience across different partners (communication service providers), channels (cellular network, Wi-Fi, Wired LAN), and access methodologies (such as 5G-NR, 4G LTE-EPC, Wi-Fi bands) in the enterprise IoT network ecosystem.
2. Secure connectivity across the IoT network (same security policy across different partners, access channels, and methodologies).
3. Convergent and holistic view of the ecosystem to the different stakeholders in the dedicated IoT network.
4. Game is free of charge to the customer. Gaming Inc charges the customer for features (high speed gaming over dedicated network slice), devices, sensors, and characters (avatars).
5. Gaming customer activity-/inactivity-based behavior for security and session management.
6. Customer usage pattern-based dynamic and enhanced authentication, authorization, and accounting (on Gaming Inc., edge or device) for catering to the different requirements in the dedicated IoT network as described earlier using the Zachman framework.
7. Content provider provides premium media content including famous proprietary profiles, avatars and their related video for the game.
8. Content provider charges Gaming Inc for the premium content as accessed by its subscribers.
9. Seamless integration of new stakeholders and enterprise in the dedicated IoT network.
10. Gaming Inc to retain customer sensitive data on its own server and not on the edge cloud provided by the service provider.
11. Sensitive information to be passed as range or state as required for the edge computing rather than the sensitive value itself.
12. Gaming Inc to have network slice with multiple communication/internet service providers, and agreements for gaming experience and charging and billing as well.
13. Convergent billing and billing as a service for the different stakeholders in the system.

Eight selected scenarios and change scenarios as discussed and developed with the various domain and IoT experts have been mentioned in Table 2. These scenarios have been written in terms of the Zachman framework.

### 5.2. C2—Architectures

As a baseline for the existing systems, a multi-access edge computing (MEC) [43,44,45] and software defined network has been studied and discussed.

Further based on the requirements of the use case and its scenarios two candidate architectures were prepared, viz. “smart AAA agent”, and “static AAA agent”. Both these architectures support a distributed, multi-agent architecture, that can intelligently take care of the requirements of the dedicated IoT network with computing being performed over the edge or at the server based on the requirement, policy, or context of execution, as depicted in Figure 2. Both of these systems and architectures derive inspiration from our previous research [8], and utilize the Zachman framework for converting the requirements into a set of authentication, authorisation, and accounting statements. They also support distributed data store and caching amongst the various agents for taking care of the sensitive data related handling to meet the specific requirements of raw data being available or not at a node and various other policy requirements. Both the architectures are quite similar except for the top two layers. The “knowledge processing layer” in both the architectures is the seat of intelligence and has an engine which governs all other components in a layer above and below.

Following is a brief explanation of the two proposed architectures:

#### 5.2.1. Smart AAA Agent Architecture

**Figure 3:**
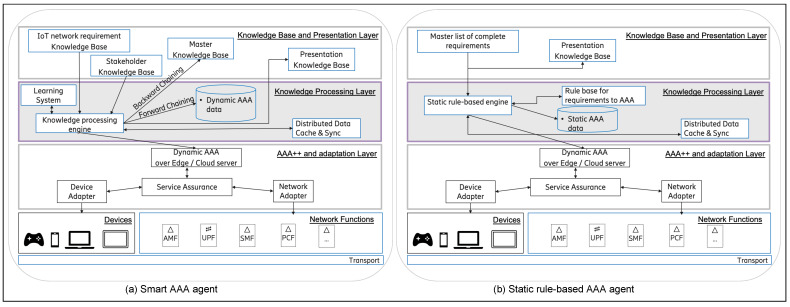
Proposed AAA agent candidate system and architecture.

The smart AAA agent architecture as depicted in Figure 3a has its smart and adaptive intelligence in the top two layers viz. “Knowledge Base and Presentation Layer” and the “Knowledge Processing Layer”. For the sake of a proof of concept and exemplification, the architecture python knowledge engine (PyKE) has been used for the artificial intelligence in the system [46,47]. PyKE uses fact-bases and rule-bases as part of the knowledge base in the system with the expert system engine processes for bringing in the intelligence in the system. This system employs a decision tree as part of the logical component to resolve all the existing requirements and policies of the system as introduced by the different stakeholders in the dedicated IoT network.

The “IoT network requirement knowledge base” is responsible for maintaining the complete knowledge base comprising of the evolving requirements of the dedicated IoT network.

The “stakeholder knowledge base” is responsible for maintaining the evolving stakeholder ecosystem and their individual policies.

“Presentation knowledge base” is responsible for maintaining the knowledge base for the different presentations required in the dedicated IoT network.

The “knowledge processing engine” is the heart of the system and it interacts with all other components in an engine and is also responsible for the coordination between the different agents. It is this component which brings about the smart and adaptive environment by intelligently allowing the assimilation of stakeholder and requirements on the go. It also supports the learning- and context-based execution and processing and supports autonomic network management. This smart and adaptive engine has the functionality to automatically assimilate the evolution and many stakeholders in the system so that they can work together. The introduction or removal of any stakeholder is processed keeping the complete system in perspective, thus smartly adapting the environment for it. Based on the context of the gaming user as well as other different stakeholders in the network, autonomic network management is accomplished in the system by smart and automatic distribution of the processing, AAA, data storage, and caching in the system based on the knowledge base defined for efficient management of the system. For example, suppose there is a policy as part of the system to store sensitive data only on the secure central server. In that case, a cache-based category is propagated on the edge automatically for quicker processing. On the other hand, without such restriction, such data could be processed at the edge for the most optimum management of resources and providing low latency. The knowledge base in this component is also responsible for the efficient monitoring of the different gaming users and the large number of stakeholders in the system in an efficient manner. Based on the policies and rules, the monitoring could be conducted on the edge or the server in the most efficient manner.

Within this component, the fact bases from the different stakeholders are assimilated into the “master knowledge base” using a backward chaining mechanism keeping into perspective the overall requirements from the dedicated IoT network. Further forward chaining is employed over the master base for ascertaining the dynamic AAA entries for taking care of the different requirements in the dedicated IoT network.

Furthermore, to introduce learning into the system a clustering based anomaly detection component (“learning system”) is introduced that learns from the usage pattern in the network and clusters into safe and unsafe time for different levels of security as per the requirement. This learning system just takes care of the requirements as identified during this research. However, the same can be enhanced with a new algorithm for any other learning required in the system, such as semi-supervised learning of the context parameters of a user based on the clustered users and reinforcement learning based on positive and negative feedback to the system.

The “distributed data cache and sync” is responsible for maintaining the cache of data between the relevant agents in the different parts of the dedicated IoT network.

The remaining components of the system are responsible for providing the service assurance to the different stakeholders via the network and device adapters.

**Figure 4:**
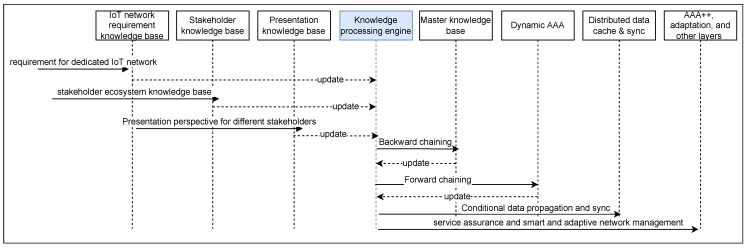
Sequence diagram depicting smart AAA agent.

The sequence diagram depicted in Figure 4 represents a sequential series of steps performed in the example scenario depicting autonomic network management. In case of a new requirement of using the heart rate of the gaming user as a contextual parameter for the game, the IoT network knowledge base is updated. A new stakeholder for a heart rate monitoring device/sensor and its knowledge base is added to the “stakeholder knowledge base". The perspective-based presentation of this heart rate to other stakeholders and view of the system to the stakeholder bringing in the heart rate measurement is added to *presentation knowledge base”. The “knowledge processing engine” has a subscription for any change to the knowledge bases and gets the corresponding update. This heart rate may be sensitive data being a health parameter of the gaming user, so it needs to be saved only on a central and secure server, with no unauthorised access. Thus the knowledge processing engine shall update the “master knowledge base” to have categorization performed on the heart rate values in the secure central server and pass only the category to the edge server near the gaming user for quick processing of the game and reducing latency in the system. Therefore, the policy of encrypted storage of sensitive parameters in the system only on the central and secure server is honoured. Yet, low latency gaming is also facilitated in the smart and adaptive environment. The knowledge processing engine, further based on these updates, generates dynamic AAA using the forward chaining mechanism and updates the “Dynamic AAA” component, which it additionally sends to the adaptation and other layers for its realization over the network and the devices. Based on the context, the “distributed data cache and sync” is also updated and data propagated. Similarly, efficient monitoring of the different stakeholders and their contextual parameters during the run time would be optimized based on the different policies and requirements in the system.

**Figure 5:**
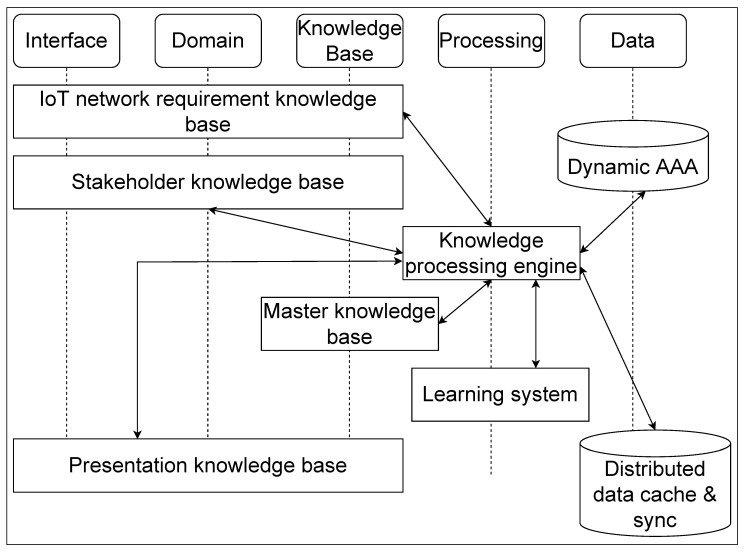
Interaction view of the main components of the smart AAA agent.

Figure 5 depicts the interaction view of the main components in the top two layers of the smart AAA agent. The leftmost “Interface” segment has three different requirements of interfacing with the admin of the system for the knowledge bases. The same three knowledge bases are the ones that construct the domain knowledge base. It starts with the first component of “IoT network requirement knowledge base”, where all the requirements of the dedicated IoT network are acquired, defined and put together. The second component “Stakeholder knowledge base” is responsible for taking the requirements of all the stakeholders together catering to all the requirements in the dedicated IoT network and their own knowledge bases of requirements and policies. This component shall have both the knowledge base corresponding to the ontology of different stakeholders and also their own policies and requirements. The third component “Presentation knowledge base” takes in the knowledge bases corresponding to the different presentation views in the system for the different stakeholders. This has to be defined in such a way that each stakeholder gets to see the correct information that they are allowed to see in a specific context.

The second vertical segment “Domain” is what comprises of all the requirements, its fulfilling stakeholders and their corresponding contextual view of the system. This comprises of the realisation of the system.

The third vertical segment “Knowledge base” is responsible for maintaining the complete repository of knowledge bases in the system in the format that is accessible to the “Knowledge processing engine”. There is a subscribe-publish pattern, which the “Knowledge processing engine” employs on the different knowledge bases to fetch, process and get any updates. This segment also contains a “Master knowledge base”, which is the post-processing knowledge base created for the system by the “Knowledge processing engine”.

The fourth vertical segment “Processing” has already been mentioned when we discussed the “Knowledge processing engine” which is the focal point of the system. It is this component that brings in the intelligence to the system. It also has a “Learning system” connected to the “Knowledge processing engine”, which caters to the various learning requirements in the system based on the inputs received from the different stakeholders and their context in the domain.

The fifth vertical segment “Data” has the two data stores. The “Dynamic AAA” contains the master knowledge base requirements translated into an enhanced AAA model based on the Zachman framework as explained in the paper earlier and exemplified in Table 1 and Table 2. The “Distributed data cache & sync” component is updated by the “Knowledge processing engine” for the distributed data that needs to be on this agent and also any cache to be maintained over the agent in the system.

#### 5.2.2. Static AAA Agent Architecture

The static AAA agent architecture as depicted in Figure 3b contains static rules for the various requirements in the dedicated IoT network. For bringing in the smart and adaptive element in such an environment, an integration is performed on a learning system to enable it to make decisions based on the different contexts.

The static AAA agent architecture as depicted in Figure 3b also has its smart and adaptive intelligence in the top two layers viz. “Knowledge Base and Presentation Layer” and the “Knowledge Processing Layer”. For the sake of a proof of concept, exemplifying the architecture, and comparison, the python knowledge engine (PyKE) has also been used in this system and architecture. This system also employs a decision tree as part of the logical component to resolve all the existing requirements and policies of the system as introduced by the different stakeholders in the dedicated IoT network. However, this does not employ the backward and forward chaining mechanisms for the intelligent assimilation of the requirements, stakeholders, and generation of the master knowledge base. Instead, it requires manual assimilation and creation of all the requirements and stakeholders in the system by experts. With large number of stakeholders and requirements this may be a herculean manual task. Furthermore, this would require redrawing the complete knowledge base with each change in the stakeholder or the requirements. Therefore, this system has a very restricted autonomic network management aspect of only altering the processing and data storage and retrieval based on predefined rules. It does not support the automatic assimilation of a new stakeholder into the system. Instead any addition or removal of a stakeholder needs to be handled with costly manual reprogramming of the master list of complete requirements. However, this system and architecture also supports a distributed architecture and can take care of the requirements of the dedicated IoT network by employing the Zachman framework’s enhanced model of AAA as depicted in the model of the “smart AAA agent”.

The “master list of complete requirements” is responsible for maintaining the knowledge fact and rule base as created manually by the experts taking all the requirements and stakeholders into perspective.

“Presentation knowledge base” is responsible for maintaining the knowledge fact and rule base for the different presentations required in the dedicated IoT network in this system as well.

The “static rule-base engine” is analogous with the “knowledge processing engine” in the other architecture. However, it lacks the intelligence for automatic assimilation of the requirements and stakeholders in the system. However, it still supports autonomic network management, albeit with the rule and fact bases being static and this system needing an integration with an external learning and intelligence system for the learning requirements.

The “rule base for requirements to AAA” is utilized by the “static rule-based engine” to generate the AAA for the complete system analogous to the other architecture.

The rest of the components behave in the same manner as in the other architecture.

Although, this system does not have intelligence of its own it is lightweight and can be integrated with any other intelligent system.

### 5.3. C3—Evaluation Results

As part of the scenario-based software architecture analysis the eight scenarios in Table 2 have been classified into six groups and evaluation assimilated from all the six experts as part of this study. The two proposed candidate architectures and systems of “smart AAA agent” and “static rule-based AAA agent” have been evaluated against the change scenario groups along with the existing network architecture comprising of multi-access edge computing (MEC) [48] and network slicing [49] technologies as defined in the 3rd generation partnership project (3GPP) standards. The participants are well versed with the existing network architecture. Therefore, an emphasis was given to explaining and discussing the proposed candidate architectures. Feedback from each participant has been recorded and a discussion and consensus was created amongst them in terms of the scenario-based software architecture analysis for all three systems. This section presents the majority consensus feedback from all participants involved in this study. Table 4 summarizes the evaluation results.

As part of the evaluation, the experts also mentioned that although a painstaking effort is required for the creation of the exhaustive knowledge base for the smart AAA agent, it helps tremendously in making the system intelligent, agile, and adaptive to any changes. In addition, a distributed system of agents is recommended, with a caching mechanism in agents at the edge to reduce any latency issues of communication in the network. The existing network architecture does not comply to the requirements. Therefore, further analysis takes into consideration only the two proposed candidate architectures.

The following is a high-level outline of the work breakdown structure (WBS) used for effort estimation:

- WBS for the Smart AAA agent (initial effort): Definition of various policies and relevant requirements for the system and its stakeholders, a task that involves Business Analysts (BA), Product Managers (PM), System Managers (SM), and System Engineers (SE). It entails: (i) BAs capturing business opportunity through workshops, and reconciling the requirements for the complete IoT network, (ii) PMs in consultations with BAs creating requirements based on the business opportunity, (iii) SMs and SEs defining and creating the knowledge-fact bases and knowledge rule base for the system with its forward and backward chaining mechanism, and its translation to technical requirements for the system, and (iv) SEs configuring the system for the corresponding knowledge bases and using test automation to secure future changes.
- WBS for the Static rule-based AAA agent (initial effort): Understanding the various policies and other relevant requirements for the system and its stakeholders. This involves similar activities as in the case of the smart AAA agent, however, for PMs, requirements design may change as the system now requires more elaborated parameters which need to be specified in the requirements. This activity can be confined to the current set of requirements and not all the policies need to be modelled into the system. It entails: (i) The same tasks for BAs as in the case of the smart AAA agent, (ii) PMs in consultation with BAs create requirements based on the business opportunity, (iii) PMs and SMs deliberate in detail the various AAA requirements from the system in the context of the above step analysis. They need to understand the policies and requirements from each stakeholder and how they fit into the larger ecosystem and create one large set of AAA requirements for the system based on the immediate needs from each of the system stakeholders, (iv) SEs configure the AAA in the system based on the analysis and inputs from the PMs and SMs as mentioned in the step above, integrate with a learning system for the classification and clustering of usage patterns, and ensure project integrations with the product base for maintainability and upgrades.

To estimate the maintenance cost of the candidate architectures, the following two indicative scenarios were chosen:

Change scenarios

1. A new sensitive piece of information such as heart rate value is now required to be acquired from the gaming user for a new feature.
2. A new service provider is introduced into the dedicated IoT network ecosystem.

WBS for the Smart AAA agent (change scenario effort):

1. BAs and PMs to understand the policies and requirements just for a new stakeholder or a policy and enlist them. It entails for Scenario 1: BA and PM introduce heart rate value in the system, and for Scenario 2: BA and PM reconcile the change with the IoT ecosystem.
2. System manager and engineer to create/update the knowledge base for the delta/change requirement. For Scenario 1: SMs incorporate the heart rate fetching feature, and SEs implement the necessary configuration and automation, and for Scenario 2: SMs introduce a new service provider and SEs implement the necessary changes in the ecosystem and automation.
3. Addition of the new/updated knowledge base in the system. For Scenario 1: system enrichment of knowledge base, and for Scenario 2: system enrichment of knowledge base.

WBS for the Static rule-based AAA agent (change scenario effort):

1. BAs and PMs to understand the policies and requirements for the new stakeholder or a policy and look at the context of the whole system and remodel the whole system. It entails for Scenario 1: BAs and PMs elaborate heart rate value and identify sensitive categorization, and for Scenario 2: BA and PM reconcile requirements with the ecosystem for which it is introduced.
2. SMs and SEs remodel the AAA for the whole system. For Scenario 1: SMs reconcile with GDPR compliance and translation of sensitive data to configuration requirements and SEs introduce necessary configuration and automation, and for Scenario 2: SMs and PMs complete the new service provider requirement’s technical translation and SEs implement the necessary configuration and automation.
3. Reconfigure the AAA for the whole system. For Scenario 1: Change as new system configuration, and for Scenario 2: Reconfiguration with the whole system
4. Integration and reconfiguration for the learning systems for both the scenarios

Table 3 presents the results of the effort estimation analysis in man days for the initial setup and the two change scenarios of both the systems, viz. “smart AAA agent” and “static AAA agent”.

## 6. Discussion and Updated Architecture

The scenario-based software architecture analysis results make it evident that the diverse requirements from the different stakeholders as part of a dedicated IoT network and its dynamically evolving nature cannot be supported with the existing telecommunications systems out of the box. The two proposed candidate systems, i.e., smart AAA agent and the static AAA agent fulfill the requirements to different degrees, with the smart AAA agent taking care of almost all the change scenarios as discussed and analyzed in the study.

**Figure 6:**
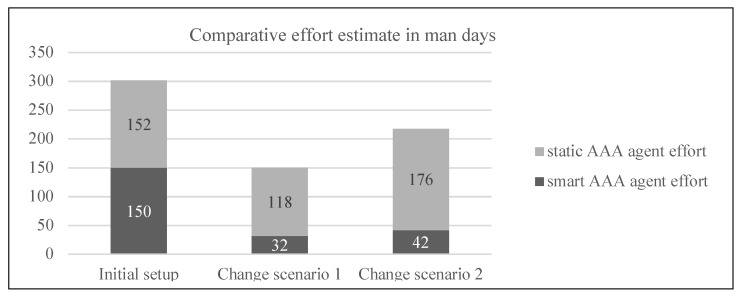
Smart AAA agent vs static AAA agent usability effort analysis.

As is evident in Figure 6, as well as in the detailed results of the effort analysis, the effort required for initial setup is similar for both the proposed candidate system and architectures. The smart AAA agent requires considerable effort for the one time creation of knowledge bases for all possible policies and requirements of the dedicated IoT network. However, the artificial intelligence in the system helps to assimilate them. Whereas the static AAA agent only needs to look at the current requirements from the system but needs an elaborate manual work of planning the AAA for the complete system. However, with each change scenario, the effort for assimilating that into the ecosystem is small in the smart AAA agent in comparison to the static AAA agent, as is evident in the difference of effort for the change scenario 1 and 2 respectively shown in Figure 6. Therefore, for a simple system with less complexity, with few stakeholders involved, and less likelihood of change scenarios, a static AAA agent is equally as good as the smart AAA agent as it is much simpler in its architecture. However, for larger systems with multiple involved stakeholders and more dynamic requirements, the smart AAA agent is a clear winner.

**Figure 7:**
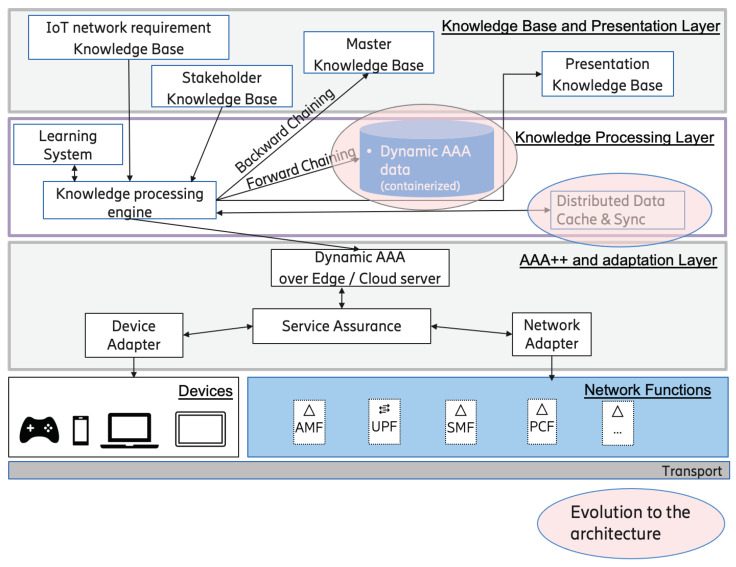
Smart AAA agent updated architecture.

The proposed candidate architecture for the smart AAA agent has been revised and updated based on the scenario-based software architecture analysis and feedback from the industry experts. The different scenarios and the distributed architecture for the smart AAA agent bring along a requirement of maintaining a cache of information at the different agents operating in the multi-agent ecosystem. Even for security purposes, it is deemed necessary to not store sensitive information at the edge. However, this information may be required in a specialized format such as a range or state and not the actual sensitive value for the execution in the dedicated IoT network. Another suggestion was containerization of the data for segregating the different stakeholders based on the access rights. Therefore, a similar addition to the architecture was conducted and is presented in Figure 7 for any future reference and use.

## 7. Conclusions

This research study has introduced a novel reference architecture and system in “smart AAA agent” for taking care of the dedicated IoT network requirements in a smart and adaptive environment.

The study begins with interviews, analysis, evolution, and presentation of an industry use case for online gaming as a representative use case for the enhanced mobile broadband spectrum. We identified three new major requirements: the need to support multiple service providers and to enable billing as a service, and billing on behalf of the use case.

The candidate reference architecture along with an alternative architecture and system were created, presented and evaluated with leading industry IoT and telecommunications domain experts. We found the Zachman framework very useful to describe the enterprise level requirements for a system. Furthermore, we found that the general model by [28] provides a systematic and streamlined approach for architectural design in industrial settings.

Several relevant scenarios have been discussed and a scenario-based software architecture analysis was performed evaluating the new smart AAA agent alongside an alternate static AAA agent and the existing telecommunication systems, thus identifying the smart AAA agent with its adaptive and intelligent capabilities as the most suitable architecture for the given use case and its scenarios.

A detailed analysis has been performed with the experts for the two proposed candidate architectures and an evaluation was performed for their usability under different circumstances. The smart AAA agent stands out as a better fit for the scenarios in contention and where there are a large number of stakeholders involved and the requirements and relationships are changing dynamically, whereas, the static AAA agent provides a lightweight system, which is good for smaller systems with more clearly defined initial requirements and lesser change scenarios later, it requires far more effort for any change scenario and introducing dynamicity to the system.

In future we can enhance the smart AAA agent with a reinforcement learning model, which can train the system quickly to derive logical decisions on its own. Furthermore, the two proposed candidate systems also need to be studied from a performance aspect while executing at different locations. This can help evolve the architecture and also provide a reference system for industry and academia for future developments.

## Figures and Tables

**Table 1:** Dynamic AAA requirements described using the Zachman framework.

	What	How	Where	Who	When	Why
Authentication	Gaming device, mobile device with gaming app, gaming customer, IoT network stakeholder	Device id, pin, password, fingerprint, face recognition, iris	Network and other zones, geographical location, auth engine	Device, customer, IoT administrator, service providers	Time, day, state of device	Security, risk mitigation, change of location and jurisdiction, & other motivations
Authorization	Device administration, premium gaming, sensitive information	Local, workflow, message queue	Location based authorization and associated workflow	Device, gaming customer, IoT admin, stakeholder	Conditional, time, day, state of device	SLA (service level agreement), service, monetization, maintenance, criticality and mitigation
Accounting	ML (machine learning) data, device data, network data	Local log, event record, workflow, message queue	On device, edge, stakeholder cloud, server	Device, user, stakeholder network, application	Continuous, conditional or need based	IoT network requirement, optimization, UX (user experience)

**Table 2:** Change scenarios for an enterprise gaming IoT network depicted in the Zachman framework model of basic interrogatives.

Quality Attributes			ID	Zachman’s Interogatives					
Performance	Functional suitability	Modifiability		What	Who	Where	How	When	Why
	✔	✔	S1	Premium Gaming Customer’s new sensitive information such as heart rate acquisition of an existing customer of a partner communication service provider (CSP) is to be done in the Gaming Inc cloud/server using the secure REST interface and following security policy when a new request is received at Gaming Inc server for bio feedback-based gaming because it is very important, sensitive and urgent information.					
✔	✔	✔	S2	Access of premium Gaming Customer sensitive information such as heart rate of an existing customer of a partner CSP is to be conducted from the Gaming Inc cloud/server using the secure REST interface and on encrypted state/range information as required by the game, rather than the original heart rate value when a new request is received at Gaming Inc server for bio feedback-based gaming because it is very important, sensitive and urgent information.					
	✔	✔	S3	Existing premium Gaming Customer Network slice information provisioning of an existing customer of Gaming Inc is to be conducted in a new partner communication service provider using the dedicated NSSAI interface when a new partner Communication Service provider onboards the partnership because it is very important, and operational information for high-speed gaming service delivery and monetization.					
	✔	✔	S4	Gaming app to require lower level of configured security for access of gaming app consumer and their device in home location using the secure REST interface when the consumer is trying to access game and has pre-registered it as home location in the system because it is important for security and ease of access in the system.					
	✔	✔	S5	Gaming app to require lowest level of configured security for access of gaming app consumer and their device in home location and the usual pattern of time for the customer using the secure REST interface when the consumer is trying to access game and has pre-registered it as home location in the system and the system has learned the usage pattern and classified it safe because it is important for security and ease of access in the system.					
	✔	✔	S6	A new SP being introduced in the dedicated IoT network by the IoT network administrator in admin office location using the secure REST interface should be performed on demand and seamlessly to allow the intelligent enterprise integration and enhancement.					
	✔	✔	S7	The event records to be sent to billing as a service provider for the consumption of media content from the new content provider just introduced in the dedicated IoT network by the IoT network administration in smart AAA agent and IoT network administration using the secure REST interface should be conducted on onboarding of a new content provider and seamlessly to allow the intelligent enterprise integration and enhancement.					
	✔	✔	S8	Event records to be split, merged or duplicated for a stakeholder having a different configuration of time zone, calendar and cycles in smart AAA agent and IoT network administration using the secure REST interface should be conducted on onboarding or any corresponding change for a stakeholder and seamlessly to allow the intelligent enterprise integration and enhancement.					

**Table 3:** Effort estimation in man days of the initial setup and the two change scenarios for the smart AAA agent and the static AAA agent.

Candidate Architecture	Tasks Based on Work Break Down Structure	Three-Point Estimation			Work Package Effort (PERT)	Risk Coverage	Total Estimated Effort in Man Days (with 68% Probability)
		*o*	*m*	*p*	*te*		
Initial/Upfront effort estimate.							
Smart AAA agent	Business analyst and project management	27	49	65	48	18	150 (±standard deviation of 4.5)
	System management and engineering tasks	20	30	40	30	15	
	Configuration and automation	19	26	35	26	13	
Static AAA agent	Business analyst and project management	16	29	38	28	18	152 (±standard deviation of 3.6)
	System management and engineering tasks	43	59	81	60	22	
	Maintainability	5	7	10	7	5	
Change scenario 1: New sensitive information such as a heart rate value is now required to be acquired from the gaming user for a new feature.							
Smart AAA agent	Business analyst and project management	5	8	10	8	3	32 (±standard deviation of 1.5)
	System management and engineering tasks	5	9	14	9	4	
	Knowledge base configuration	3	5	8	5	2	
Static AAA agent	Business analyst and project management	16	20	24	20	10	118 (±standard deviation of 2.8)
	System management and engineering tasks	23	30	37	30	13	
	Configuration	10	15	18	15	8	
	Integration and reinforcement	10	15	18	15	8	
Change scenario 2: A new service provider is introduced into the dedicated IoT network ecosystem.							
Smart AAA agent	Business analyst and project management	7	10	12	10	4	42 (±standard deviation of 1.4)
	System management and engineering tasks	11	15	18	15	6	
	Knowledge base configuration	3	5	8	5	2	
Static AAA agent	Business analyst and project management	20	24	30	24	11	176 (±standard deviation of 3.7)
	System management and engineering tasks	40	51	60	51	23	
	Configuration	20	25	30	25	11	
	Integration and reinforcement	15	20	25	20	11	

**Table 4:** Evaluation results and classification based on change scenarios.

Scenario	Existing Network Architecture	Static Rule-Based AAA Agent	Smart AAA Agent for Dedicated IoT Network
S1	**Not compliant**, requires explicit configuration and adaptation in the system	**Partially compliant**, with some level of classification of data into sensitivity categories and explicit configuration for the same	**Compliant**, with the intelligent system performing the machine reasonings based on the configured knowledge base
S2	**Not compliant**, requires integration with an intelligent system that can classify the data into the required format	**Partially compliant**, requires integration with an intelligent system that can classify the data into the required format	**Compliant**, with the intelligent system performing the machine reasonings based on the configured knowledge base
S3 & S6	**Not compliant**. It requires huge effort for integration with other networks and creating a centralized “Home Subscriber server” kind of system for the whole dedicated IoT network	**Partially compliant**, requires explicit configuration of rules for the new stakeholder/communication service provider	**Compliant**, with addition of the knowledge base for the new service provider and the intelligent system assimilates it into the existing knowledge base for the system
S4 & S5	**Not compliant**, requires integration to an intelligent learning system	**Partially compliant**, and requires explicit rules to be configured and integration to a learning system	**Compliant**, with the intelligent learning system and knowledge rule base as an integral part of the system
S7	**Not compliant**	**Partially complaint**, requires an explicit configuration of rules for the new content provider introduced in the system	**Compliant**, with the configured knowledge base in the system taking care of the requirement
S8	**Not compliant**, requires integration with a mediation system or some other custom solution for the same	**Compliant**, however requires explicit configuration of accounting rules for the new stakeholder or the requirements	**Compliant**, with the configured knowledge base in the system taking care of the requirement

## Abbreviations

The following abbreviations are used in this manuscript:

IoT	Internet of Things
AAA	Authentication, authorization, and accounting
MEC	Multi-access Edge Computing
UX	User experience
ML	Machine learning
SLA	Service Level Agreement
SAAM	Scenario-based Architecture Analysis Method
PERT	Program Evaluation and Review Technique
BSS	Business Support System
PMBOK	Project Management Book of Knowledge
WBS	Work Breakdown Structure
S-NSSAI	Single Network Slice Selection Assistance Information
5G-NR	5G New Radio
LTE	Long-Term Evolution
EPC	Evolved Packet Core
CSP	Communication Service Provider
REST	Representational State Transfer
SP	Service Provider
PyKE	Python Knowledge Engine
3GPP	3rd Generation Partnership Project
BA	Business Analyst
PM	Product Manager
SM	System Manager
SE	System Engineer
GDPR	EU General Data Protection Regulation
